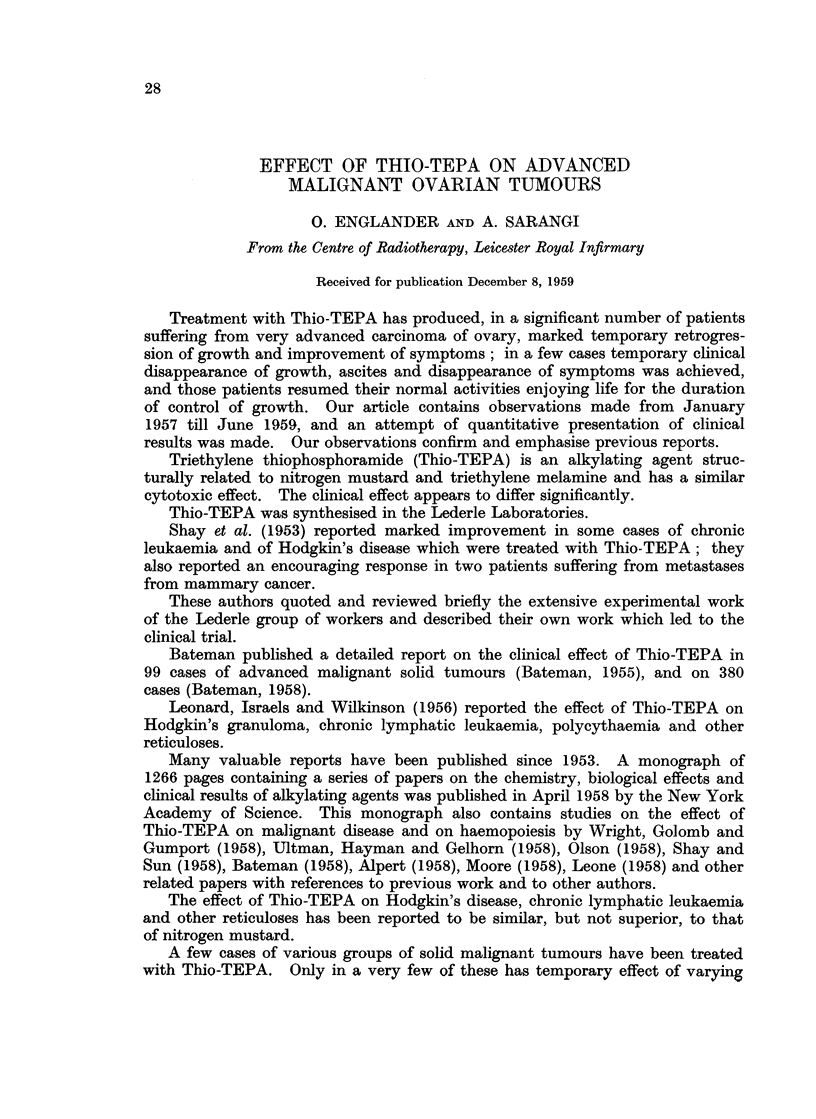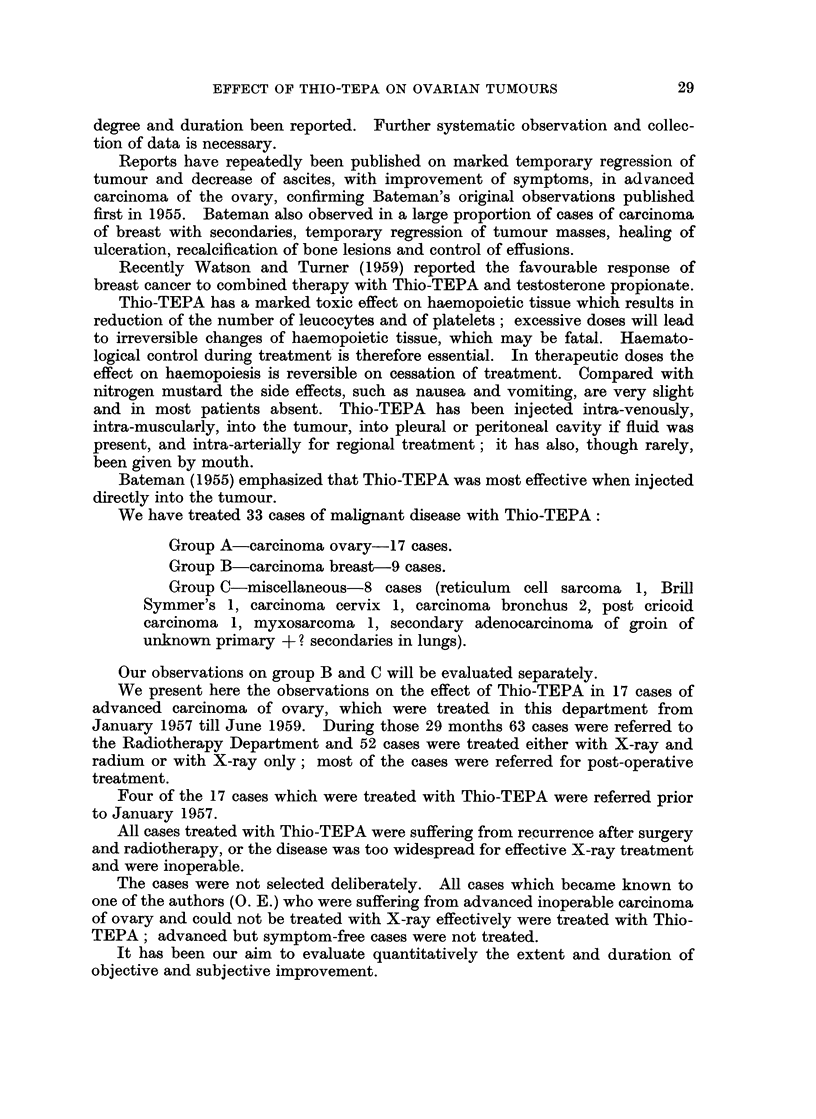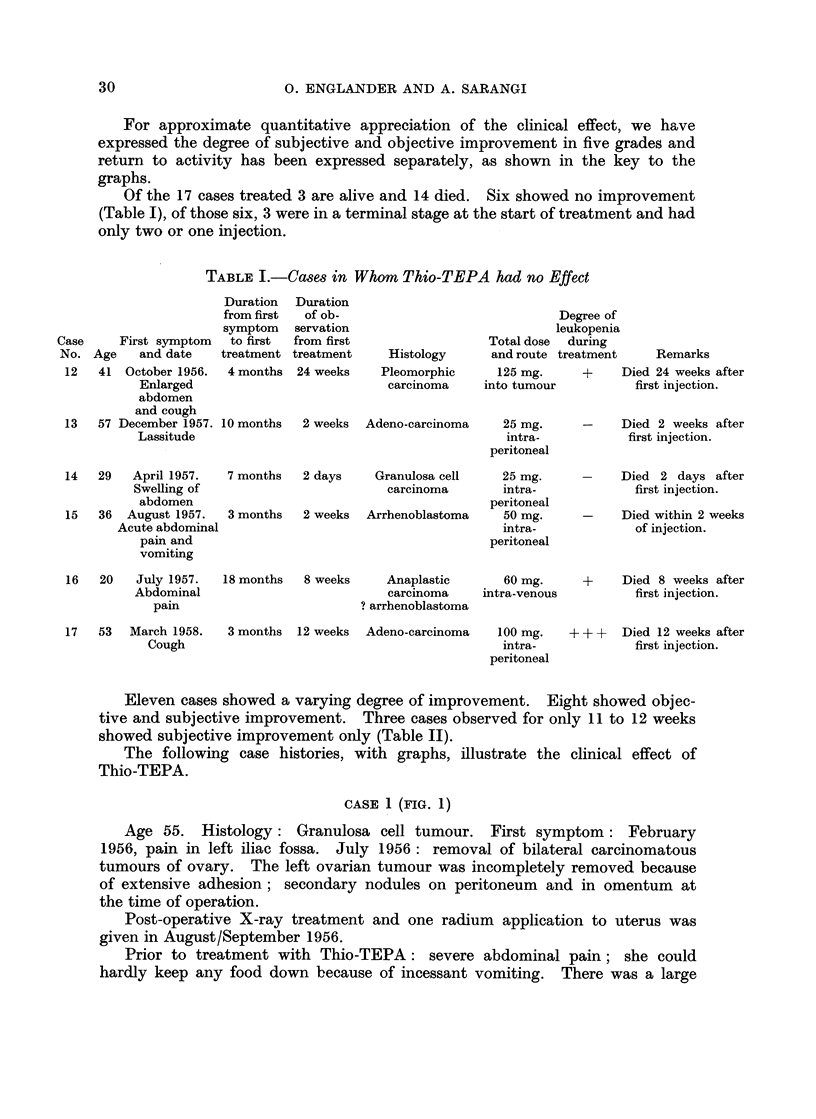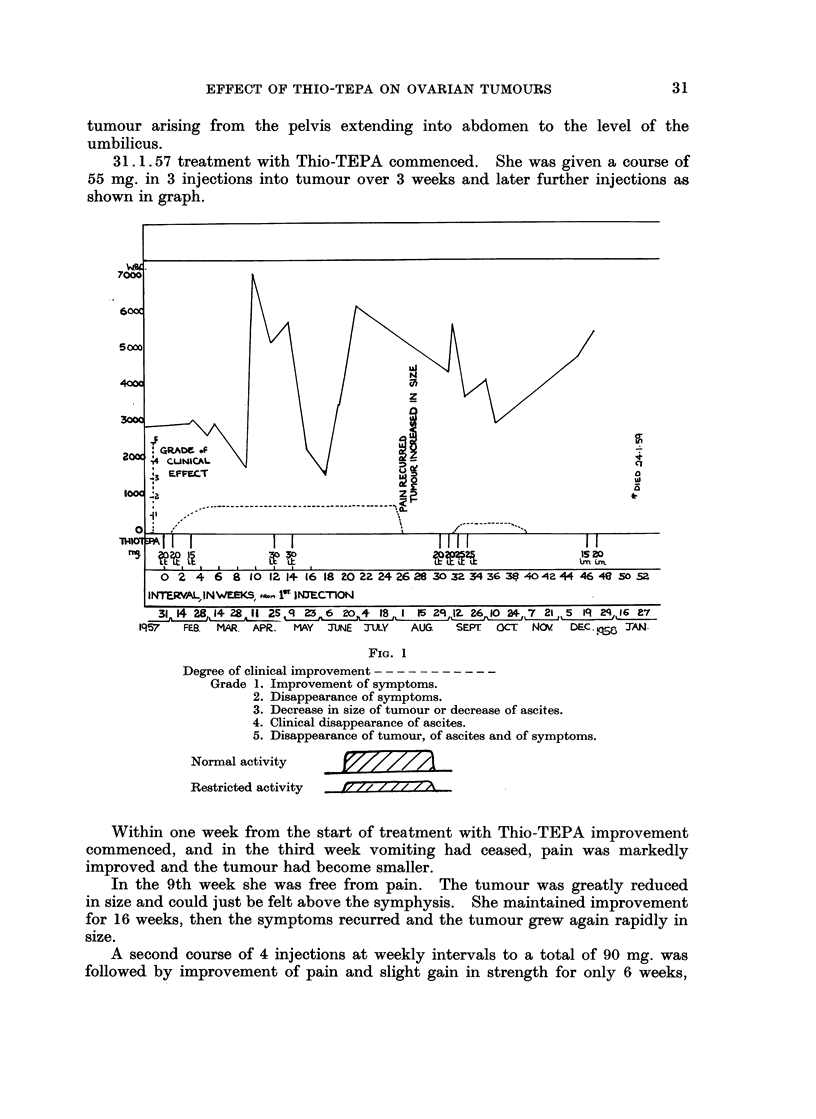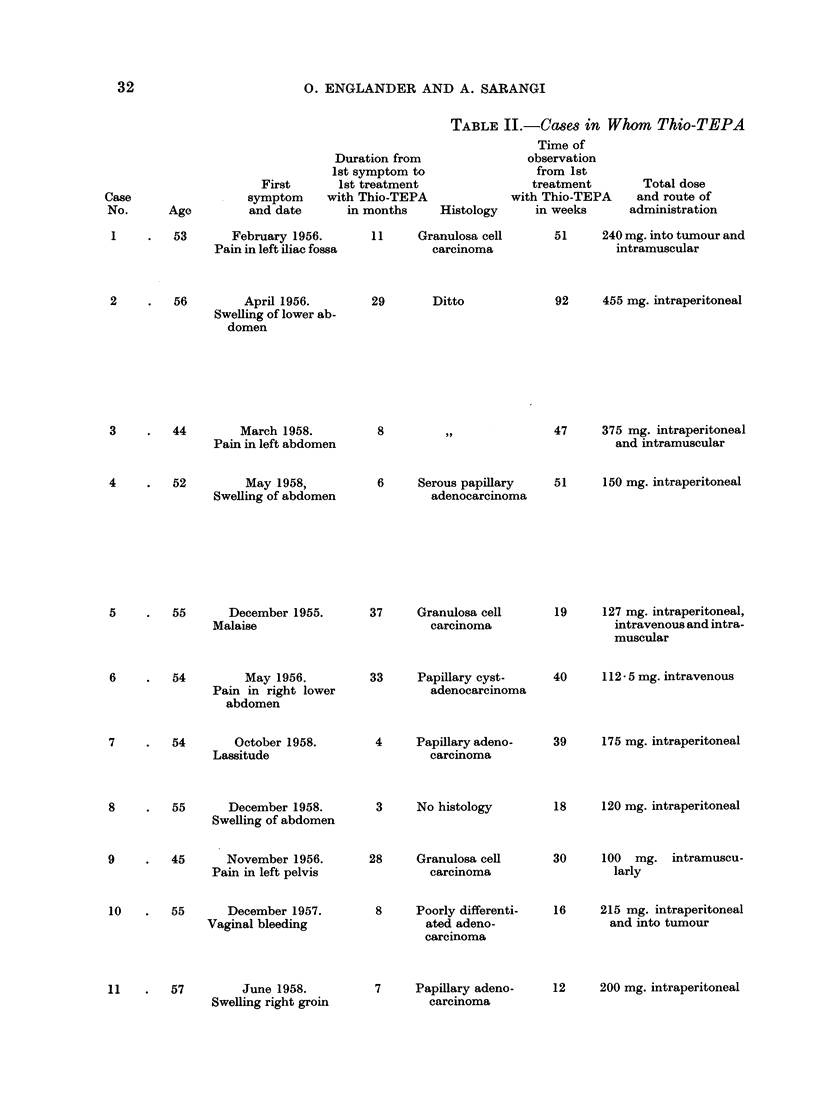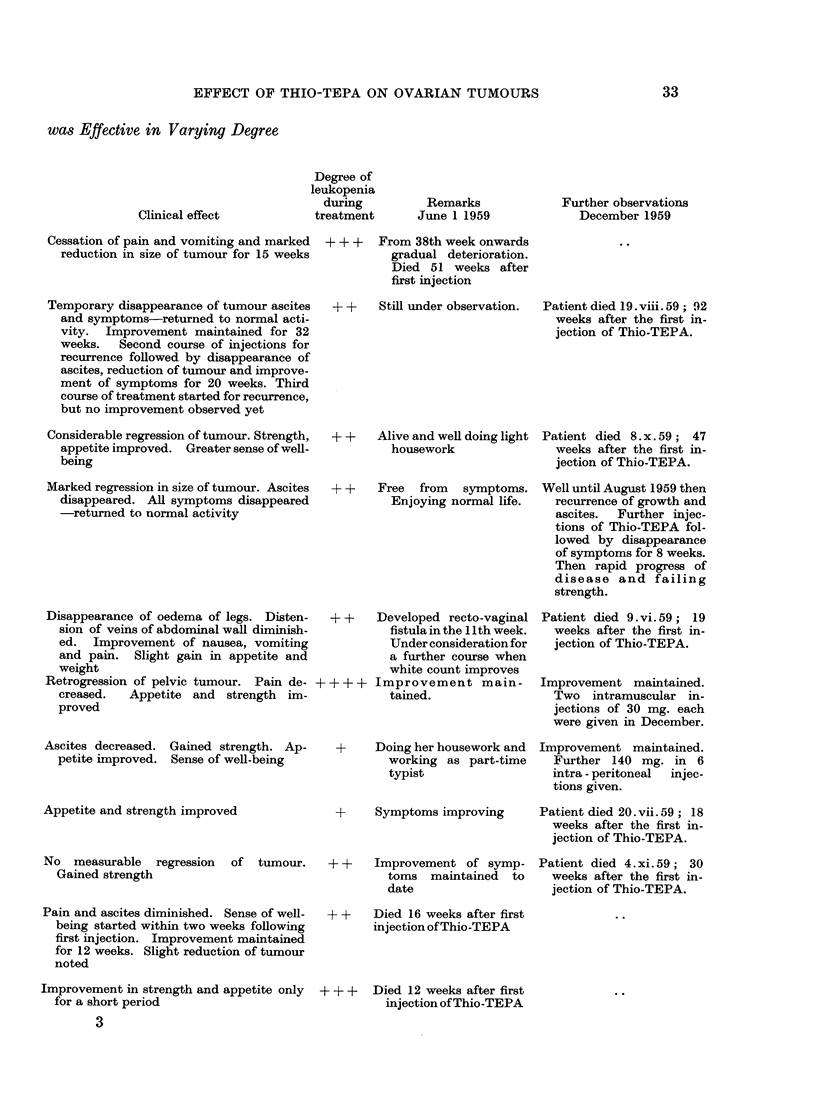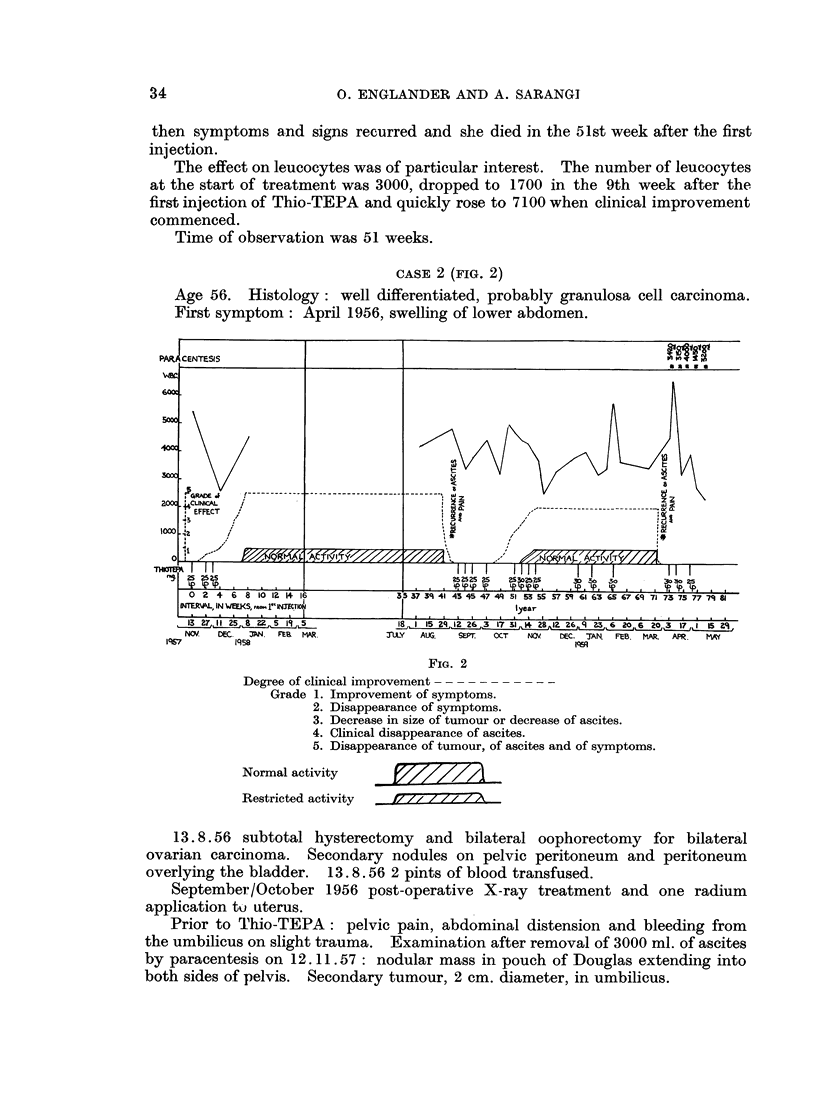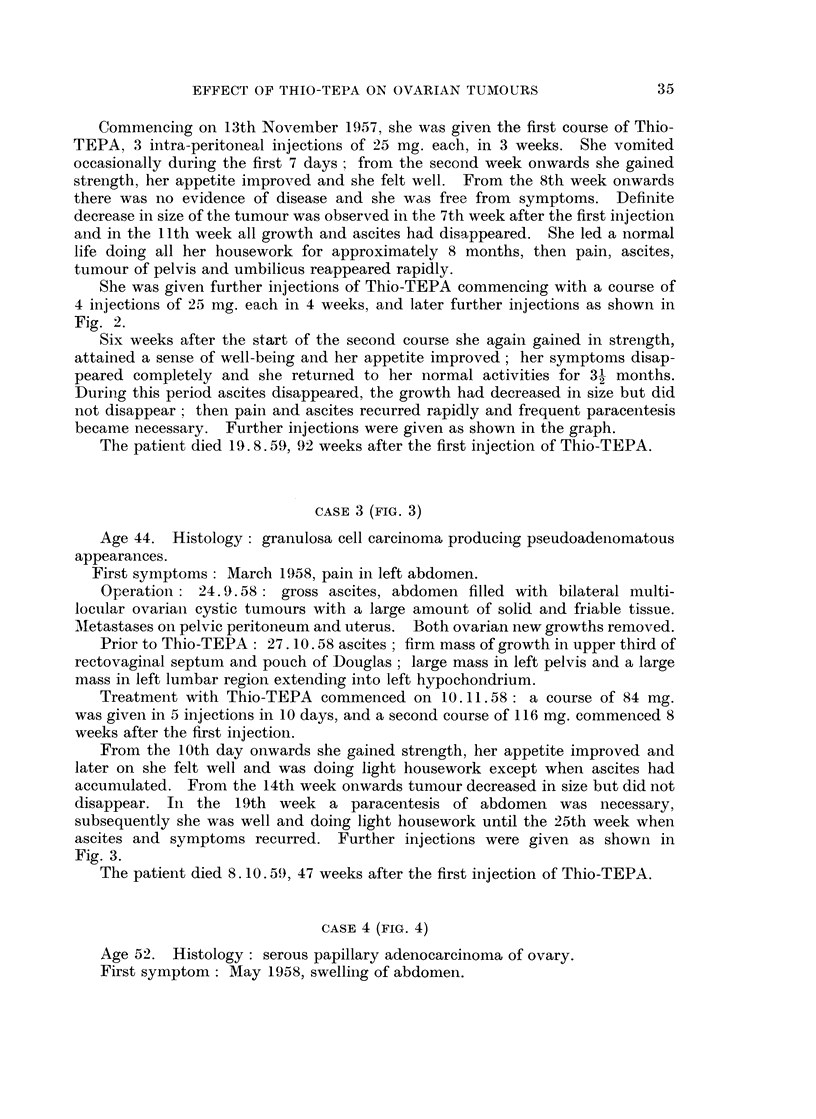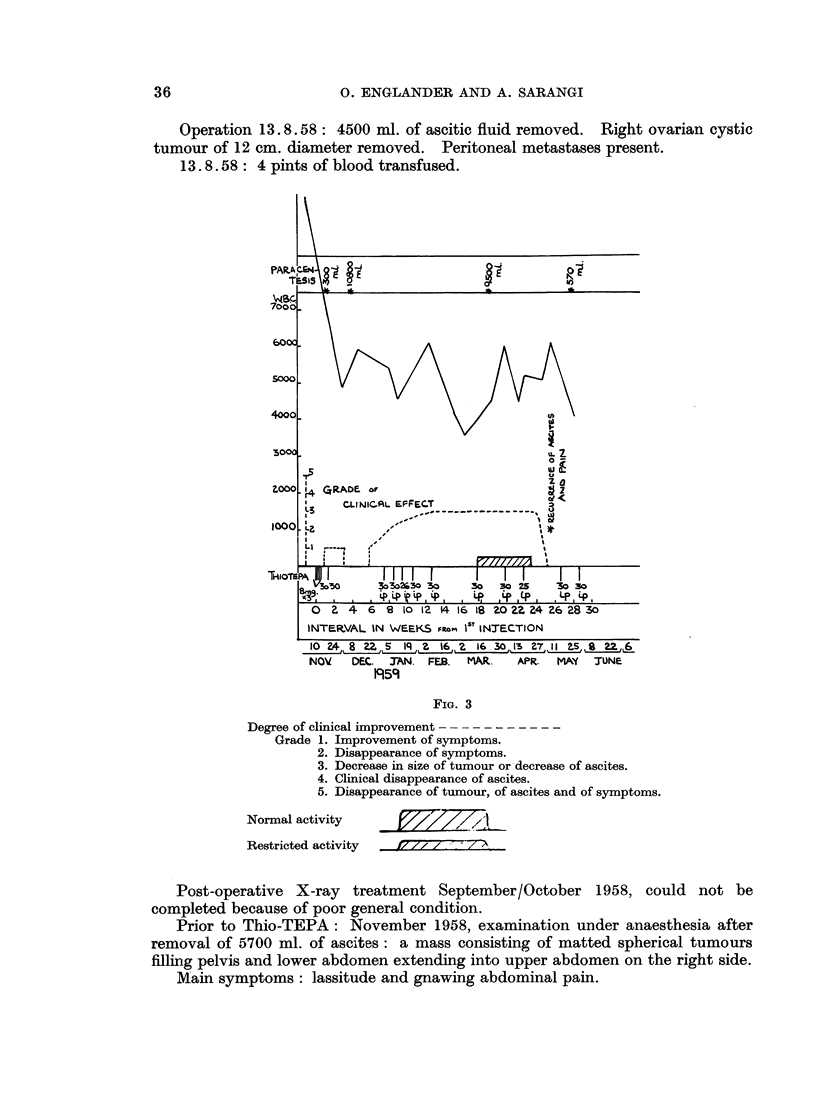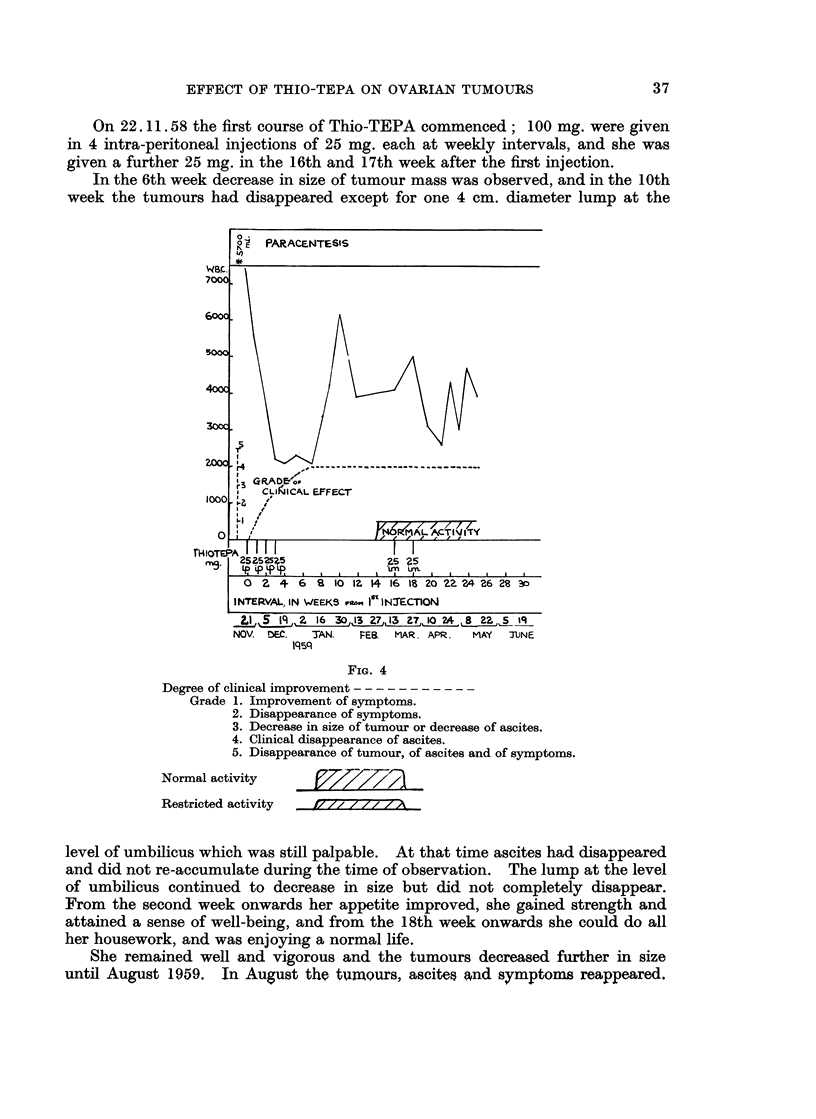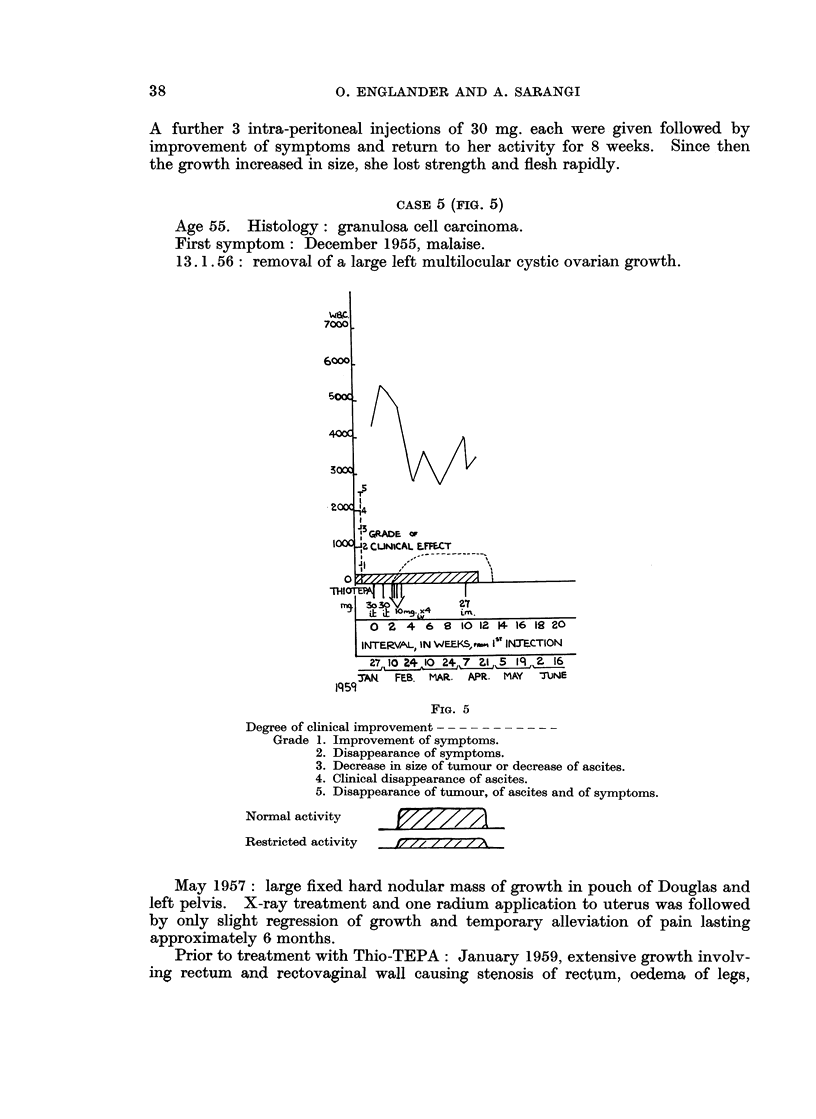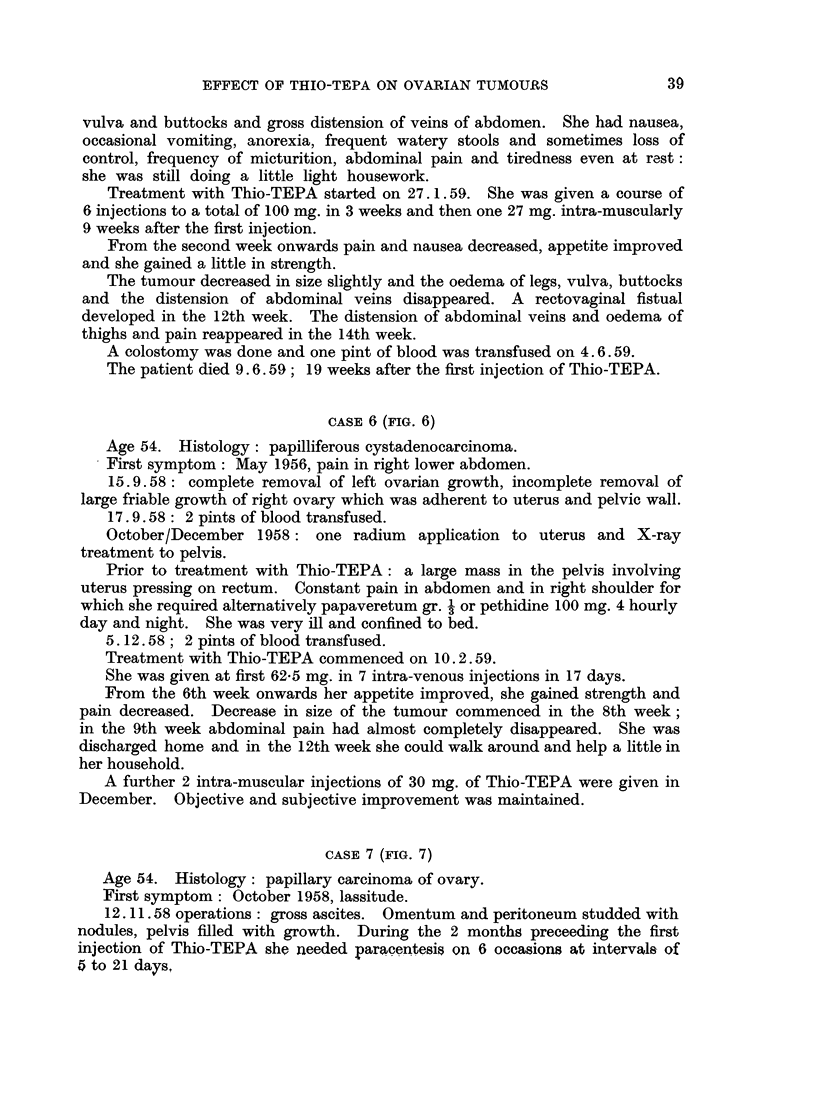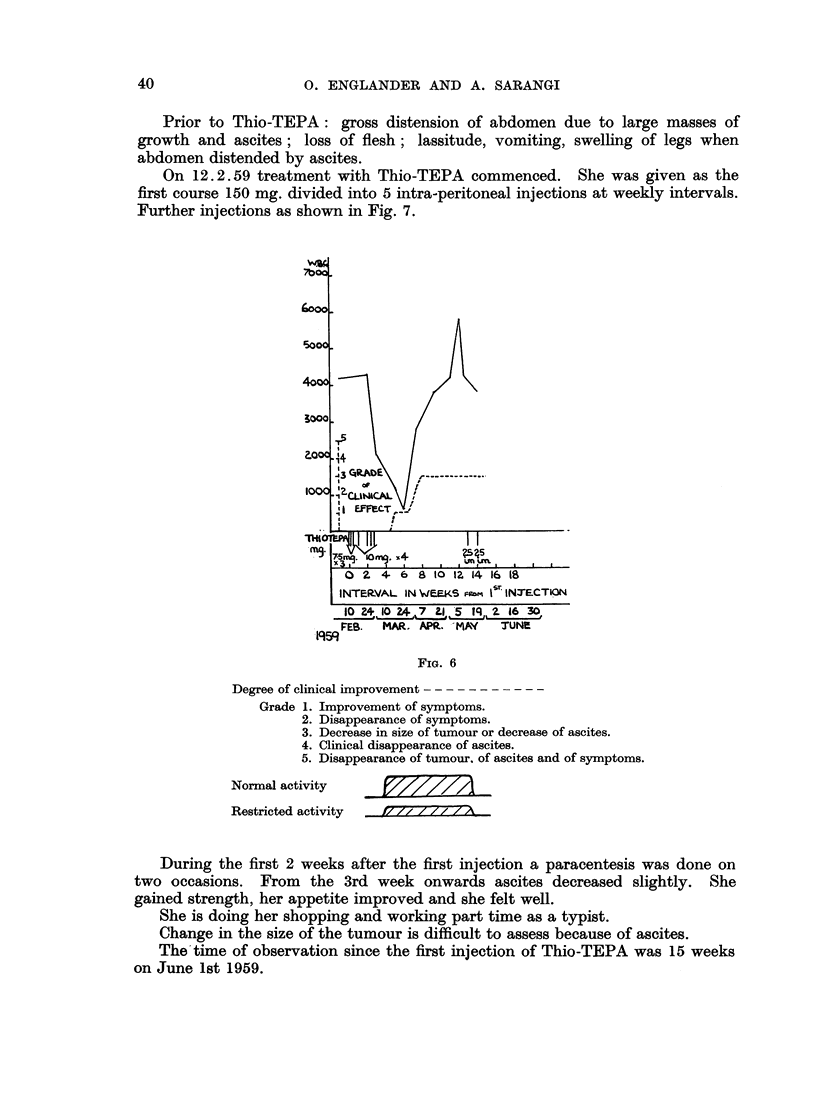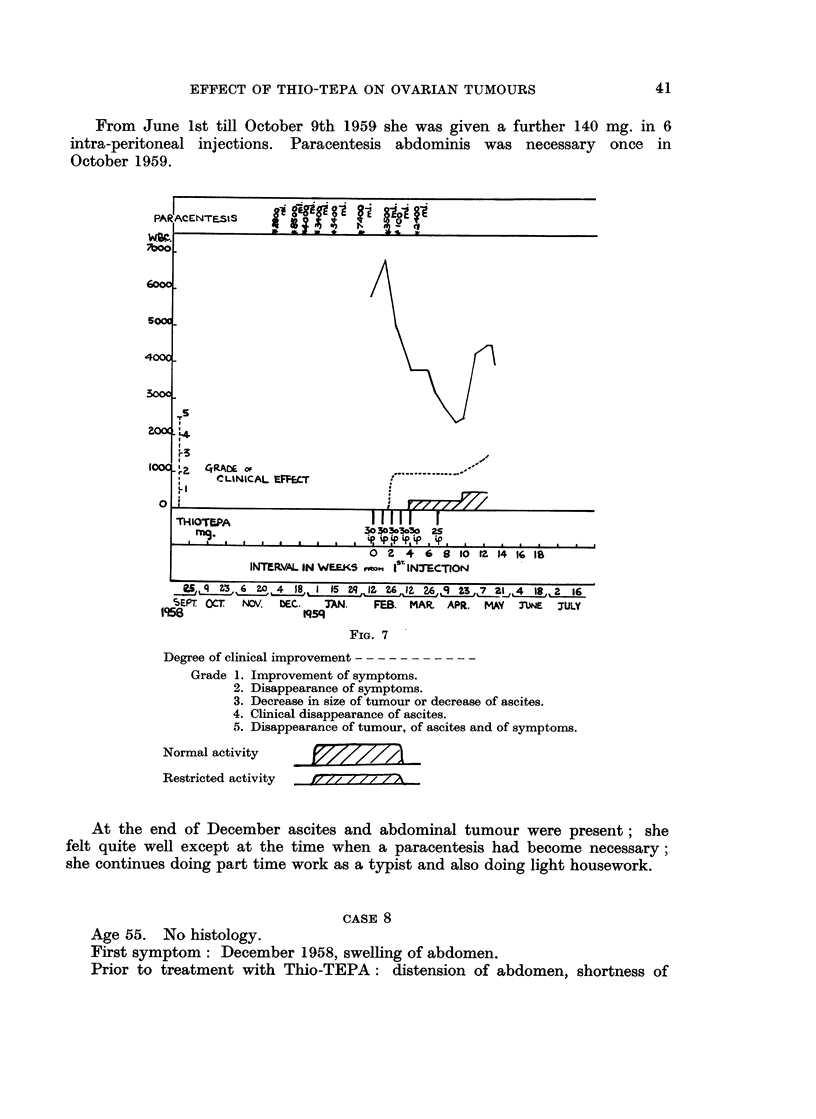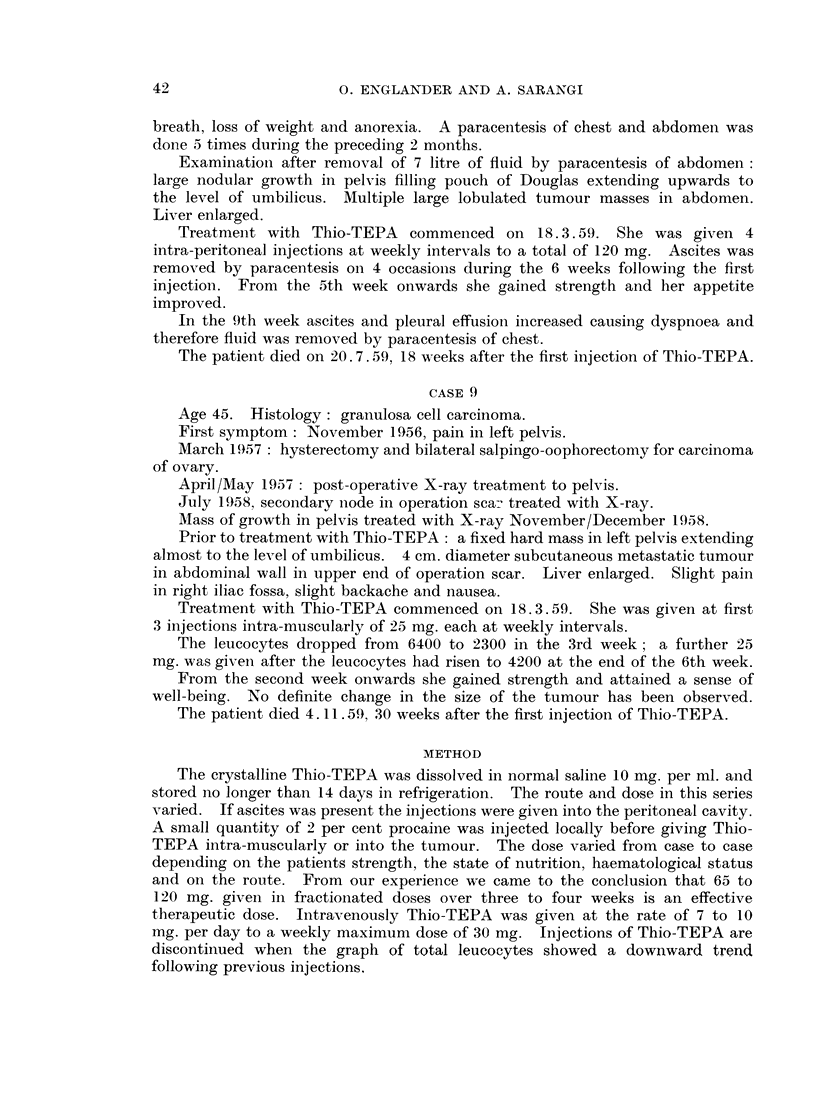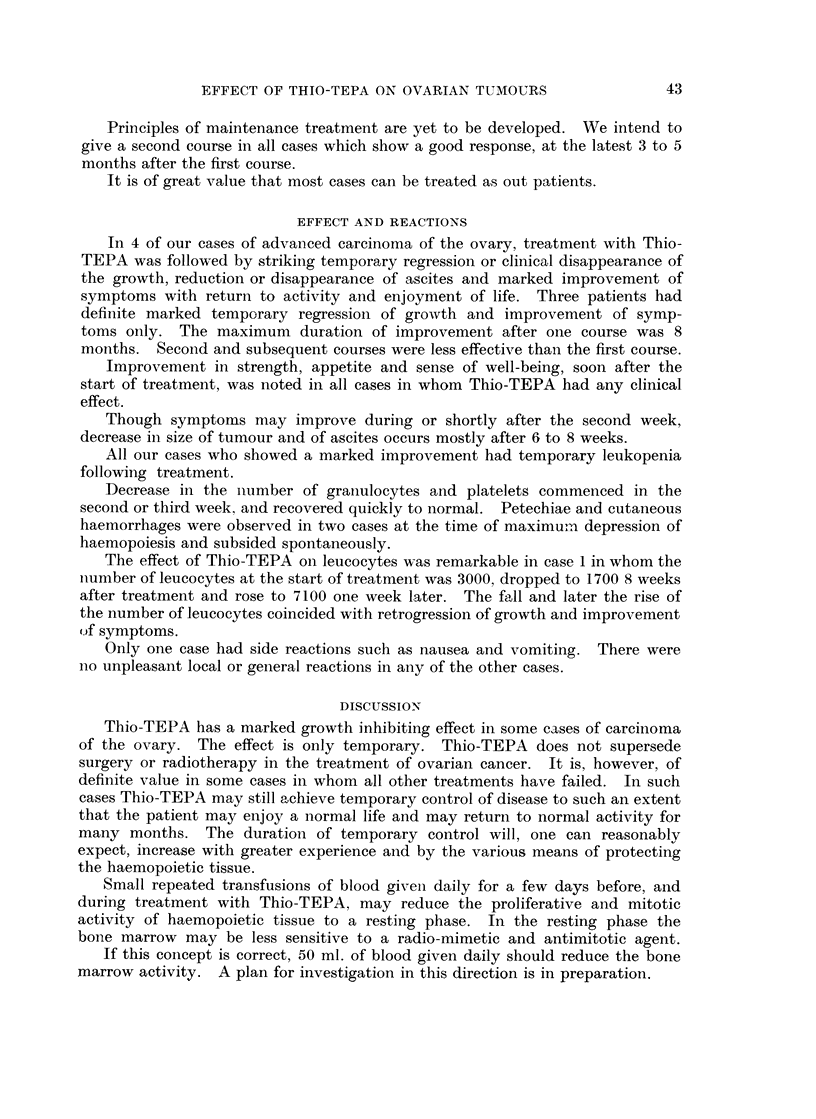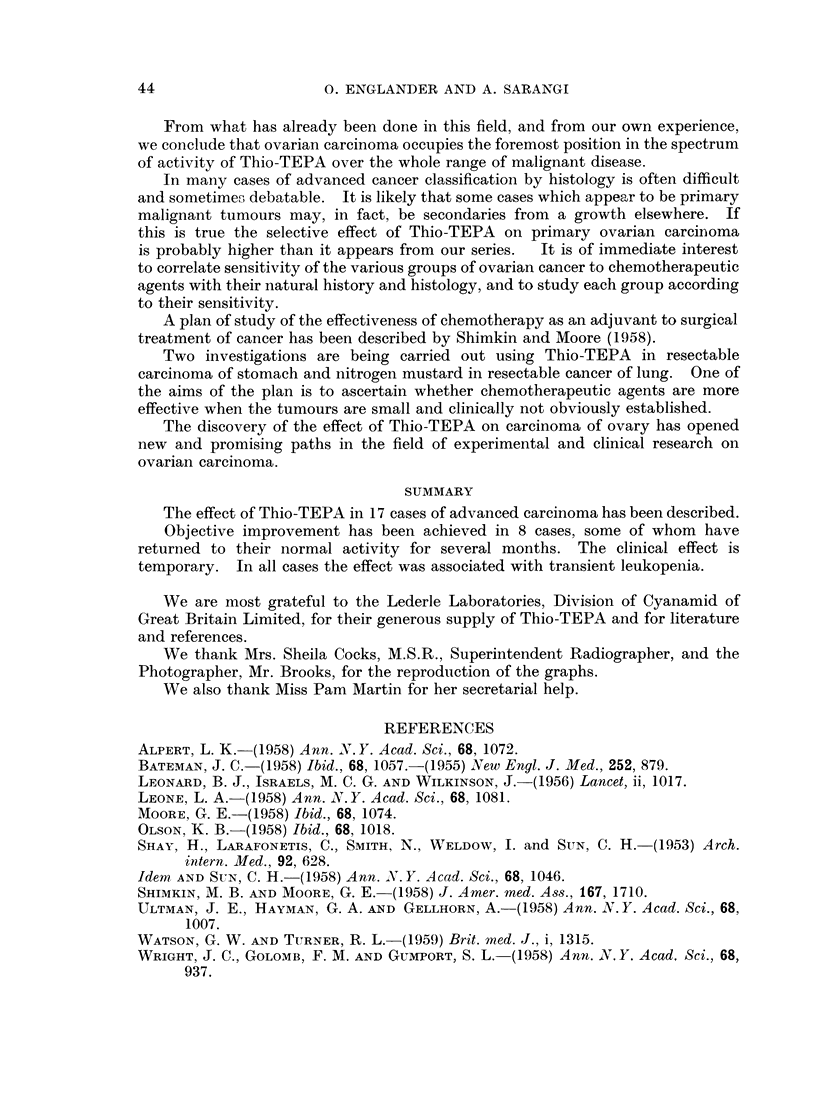# Effect of Thio-TEPA on Advanced Malignant Ovarian Tumours

**DOI:** 10.1038/bjc.1960.4

**Published:** 1960-03

**Authors:** O. Englander, A. Sarangi


### EFFECT OF THIO-TEPA ON ADVANCED MALIGNANT OVARIAN TUMOURS

O. ENGLANDER AND A. SARANGI

*From the Centre of Radiotherapy, Leicester Royal Infirmary*

Received for publication December 8, 1959

Treatment with Thio-TEPA has produced, in a significant number of patients suffering from very advanced carcinoma of ovary, marked temporary retrogression of growth and improvement of symptoms ; in a few cases temporary clinical disappearance of growth, ascites and disappearance of symptoms was achieved, and those patients resumed their normal activities enjoying life for the duration of control of growth. Our article contains observations made from January 1957 till June 1959, and an attempt of quantitative presentation of clinical results was made. Our observations confirm and emphasise previous reports.

Triethylene thiophosphoramide (Thio-TEPA) is an alkylating agent structurally related to nitrogen mustard and triethylene melamine and has a similar cytotoxic effect. The clinical effect appears to differ significantly.

Thio-TEPA was synthesised in the Lederle Laboratories.

Shay *et al.* (1953) reported marked improvement in some cases of chronic leukaemia and of Hodgkin's disease which were treated with Thio-TEPA ; they also reported an encouraging response in two patients suffering from metastases from mammary cancer.

These authors quoted and reviewed briefly the extensive experimental work of the Lederle group of workers and described their own work which led to the clinical trial.

Bateman published a detailed report on the clinical effect of Thio-TEPA in 99 cases of advanced malignant solid tumours (Bateman, 1955), and on 380 cases (Bateman, 1958).

Leonard, Israels and Wilkinson (1956) reported the effect of Thio-TEPA on Hodgkin's granuloma, chronic lymphatic leukaemia, polycythaemia and other reticuloses.

Many valuable reports have been published since 1953. A monograph of 1266 pages containing a series of papers on the chemistry, biological effects and clinical results of alkylating agents was published in April 1958 by the New York Academy of Science. This monograph also contains studies on the effect of Thio-TEPA on malignant disease and on haemopoiesis by Wright, Golomb and Gumport (1958), Ultman, Hayman and Gelhorn (1958), Olson (1958), Shay and Sun (1958), Bateman (1958), Alpert (1958), Moore (1958), Leone (1958) and other related papers with references to previous work and to other authors.

The effect of Thio-TEPA on Hodgkin's disease, chronic lymphatic leukaemia and other reticuloses has been reported to be similar, but not superior, to that of nitrogen mustard.

A few cases of various groups of solid malignant tumours have been treated with Thio-TEPA. Only in a very few of these has temporary effect of varying degree and duration been reported. Further systematic observation and collection of data is necessary.

Reports have repeatedly been published on marked temporary regression of tumour and decrease of ascites, with improvement of symptoms, in advanced carcinoma of the ovary, confirming Bateman's original observations published first in 1955. Bateman also observed in a large proportion of cases of carcinoma of breast with secondaries, temporary regression of tumour masses, healing of ulceration, recalcification of bone lesions and control of effusions.

Recently Watson and Turner (1959) reported the favourable response of breast cancer to combined therapy with Thio-TEPA and testosterone propionate.

Thio-TEPA has a marked toxic effect on haemopoietic tissue which results in reduction of the number of leucocytes and of platelets ; excessive doses will lead to irreversible changes of haemopoietic tissue, which may be fatal. Haematological control during treatment is therefore essential. In therapeutic doses the effect on haemopoiesis is reversible on cessation of treatment. Compared with nitrogen mustard the side effects, such as nausea and vomiting, are very slight and in most patients absent. Thio-TEPA has been injected intra-venously, intra-muscularly, into the tumour, into pleural or peritoneal cavity if fluid was present, and intra-arterially for regional treatment ; it has also, though rarely, been given by mouth.

Bateman (1955) emphasized that Thio-TEPA was most effective when injected directly into the tumour.

We have treated 33 cases of malignant disease with Thio-TEPA :

Group A—carcinoma ovary—17 cases.

Group B—carcinoma breast—9 cases.

Group C—miscellaneous—8 cases (reticulum cell sarcoma 1, Brill Symmer's 1, carcinoma cervix 1, carcinoma bronchus 2, post cricoid carcinoma 1, myxosarcoma 1, secondary adenocarcinoma of groin of unknown primary +? secondaries in lungs).

Our observations on group B and C will be evaluated separately.

We present here the observations on the effect of Thio-TEPA in 17 cases of advanced carcinoma of ovary, which were treated in this department from January 1957 till June 1959. During those 29 months 63 cases were referred to the Radiotherapy Department and 52 cases were treated either with X-ray and radium or with X-ray only ; most of the cases were referred for post-operative treatment.

Four of the 17 cases which were treated with Thio-TEPA were referred prior to January 1957.

All cases treated with Thio-TEPA were suffering from recurrence after surgery and radiotherapy, or the disease was too widespread for effective X-ray treatment and were inoperable.

The cases were not selected deliberately. All cases which became known to one of the authors (O. E.) who were suffering from advanced inoperable carcinoma of ovary and could not be treated with X-ray effectively were treated with Thio-TEPA ; advanced but symptom-free cases were not treated.

It has been our aim to evaluate quantitatively the extent and duration of objective and subjective improvement.

For approximate quantitative appreciation of the clinical effect, we have expressed the degree of subjective and objective improvement in five grades and return to activity has been expressed separately, as shown in the key to the graphs.

Of the 17 cases treated 3 are alive and 14 died. Six showed no improvement (Table I), of those six, 3 were in a terminal stage at the start of treatment and had only two or one injection.

TABLE I.—*Cases in Whom Thio-TEPA had no Effect*

| Case No. | Age | First symptom and date | Duration from first symptom to first treatment | Duration of observation from first treatment | Histology | Total dose and route | Degree of leukopenia during treatment | Remarks |
|---|---|---|---|---|---|---|---|---|
| 12 | 41 | October 1956. Enlarged abdomen and cough | 4 months | 24 weeks | Pleomorphic carcinoma | 125 mg. into tumour | + | Died 24 weeks after first injection. |
| 13 | 57 | December 1957. Lassitude | 10 months | 2 weeks | Adeno-carcinoma | 25 mg. intra-peritoneal | — | Died 2 weeks after first injection. |
| 14 | 29 | April 1957. Swelling of abdomen | 7 months | 2 days | Granulosa cell carcinoma | 25 mg. intra-peritoneal | — | Died 2 days after first injection. |
| 15 | 36 | August 1957. Acute abdominal pain and vomiting | 3 months | 2 weeks | Arrhenoblastoma | 50 mg. intra-peritoneal | — | Died within 2 weeks of injection. |
| 16 | 20 | July 1957. Abdominal pain | 18 months | 8 weeks | Anaplastic carcinoma ? arrhenoblastoma | 60 mg. intra-venous | + | Died 8 weeks after first injection. |
| 17 | 53 | March 1958. Cough | 3 months | 12 weeks | Adeno-carcinoma | 100 mg. intra-peritoneal | +++ | Died 12 weeks after first injection. |

Eleven cases showed a varying degree of improvement. Eight showed objective and subjective improvement. Three cases observed for only 11 to 12 weeks showed subjective improvement only (Table II).

The following case histories, with graphs, illustrate the clinical effect of Thio-TEPA.

### CASE 1 (FIG. 1)

Age 55. Histology: Granulosa cell tumour. First symptom: February 1956, pain in left iliac fossa. July 1956: removal of bilateral carcinomatous tumours of ovary. The left ovarian tumour was incompletely removed because of extensive adhesion; secondary nodules on peritoneum and in omentum at the time of operation.

Post-operative X-ray treatment and one radium application to uterus was given in August/September 1956.

Prior to treatment with Thio-TEPA: severe abdominal pain; she could hardly keep any food down because of incessant vomiting. There was a large tumour arising from the pelvis extending into abdomen to the level of the umbilicus.

31.1.57 treatment with Thio-TEPA commenced. She was given a course of 55 mg. in 3 injections into tumour over 3 weeks and later further injections as shown in graph.

Fig. 1

Degree of clinical improvement – – – – – – – – – – – –
Grade 1. Improvement of symptoms.
2. Disappearance of symptoms.
3. Decrease in size of tumour or decrease of ascites.
4. Clinical disappearance of ascites.
5. Disappearance of tumour, of ascites and of symptoms.

Normal activity 

Restricted activity 

Within one week from the start of treatment with Thio-TEPA improvement commenced, and in the third week vomiting had ceased, pain was markedly improved and the tumour had become smaller.

In the 9th week she was free from pain. The tumour was greatly reduced in size and could just be felt above the symphysis. She maintained improvement for 16 weeks, then the symptoms recurred and the tumour grew again rapidly in size.

A second course of 4 injections at weekly intervals to a total of 90 mg. was followed by improvement of pain and slight gain in strength for only 6 weeks,

TABLE II.—*Cases in Whom Thio-TEPA*

| Case No. | Age | First symptom and date | Duration from 1st symptom to 1st treatment with Thio-TEPA in months | Histology | Time of observation from 1st treatment with Thio-TEPA in weeks | Total dose and route of administration |
|---|---|---|---|---|---|---|
| 1 . | 53 | February 1956. Pain in left iliac fossa | 11 | Granulosa cell carcinoma | 51 | 240 mg. into tumour and intramuscular |
| 2 . | 56 | April 1956. Swelling of lower abdomen | 29 | Ditto | 92 | 455 mg. intraperitoneal |
| 3 . | 44 | March 1958. Pain in left abdomen | 8 | ,, | 47 | 375 mg. intraperitoneal and intramuscular |
| 4 . | 52 | May 1958, Swelling of abdomen | 6 | Serous papillary adenocarcinoma | 51 | 150 mg. intraperitoneal |
| 5 . | 55 | December 1955. Malaise | 37 | Granulosa cell carcinoma | 19 | 127 mg. intraperitoneal, intravenous and intramuscular |
| 6 . | 54 | May 1956. Pain in right lower abdomen | 33 | Papillary cystadenocarcinoma | 40 | 112·5 mg. intravenous |
| 7 . | 54 | October 1958. Lassitude | 4 | Papillary adenocarcinoma | 39 | 175 mg. intraperitoneal |
| 8 . | 55 | December 1958. Swelling of abdomen | 3 | No histology | 18 | 120 mg. intraperitoneal |
| 9 . | 45 | November 1956. Pain in left pelvis | 28 | Granulosa cell carcinoma | 30 | 100 mg. intramuscularly |
| 10 . | 55 | December 1957. Vaginal bleeding | 8 | Poorly differentiated adenocarcinoma | 16 | 215 mg. intraperitoneal and into tumour |
| 11 . | 57 | June 1958. Swelling right groin | 7 | Papillary adenocarcinoma | 12 | 200 mg. intraperitoneal |

*was Effective in Varying Degree*

| Clinical effect | Degree of leukopenia during treatment | Remarks June 1 1959 | Further observations December 1959 |
|---|---|---|---|
| Cessation of pain and vomiting and marked reduction in size of tumour for 15 weeks | +++ | From 38th week onwards gradual deterioration. Died 51 weeks after first injection | .. |
| Temporary disappearance of tumour ascites and symptoms—returned to normal activity. Improvement maintained for 32 weeks. Second course of injections for recurrence followed by disappearance of ascites, reduction of tumour and improvement of symptoms for 20 weeks. Third course of treatment started for recurrence, but no improvement observed yet | ++ | Still under observation. | Patient died 19.viii.59 ; 92 weeks after the first injection of Thio-TEPA. |
| Considerable regression of tumour. Strength, appetite improved. Greater sense of well-being | ++ | Alive and well doing light housework | Patient died 8.x.59 ; 47 weeks after the first injection of Thio-TEPA. |
| Marked regression in size of tumour. Ascites disappeared. All symptoms disappeared —returned to normal activity | ++ | Free from symptoms. Enjoying normal life. | Well until August 1959 then recurrence of growth and ascites. Further injections of Thio-TEPA followed by disappearance of symptoms for 8 weeks. Then rapid progress of disease and failing strength. |
| Disappearance of oedema of legs. Distension of veins of abdominal wall diminished. Improvement of nausea, vomiting and pain. Slight gain in appetite and weight | ++ | Developed recto-vaginal fistula in the 11th week. Under consideration for a further course when white count improves | Patient died 9.vi.59 ; 19 weeks after the first injection of Thio-TEPA. |
| Retrogression of pelvic tumour. Pain decreased. Appetite and strength improved | ++++ | Improvement maintained. | Improvement maintained. Two intramuscular injections of 30 mg. each were given in December. |
| Ascites decreased. Gained strength. Appetite improved. Sense of well-being | + | Doing her housework and working as part-time typist | Improvement maintained. Further 140 mg. in 6 intra-peritoneal injections given. |
| Appetite and strength improved | + | Symptoms improving | Patient died 20.vii.59 ; 18 weeks after the first injection of Thio-TEPA. |
| No measurable regression of tumour. Gained strength | ++ | Improvement of symptoms maintained to date | Patient died 4.xi.59 ; 30 weeks after the first injection of Thio-TEPA. |
| Pain and ascites diminished. Sense of well-being started within two weeks following first injection. Improvement maintained for 12 weeks. Slight reduction of tumour noted | ++ | Died 16 weeks after first injection of Thio-TEPA | .. |
| Improvement in strength and appetite only for a short period | +++ | Died 12 weeks after first injection of Thio-TEPA | .. |

then symptoms and signs recurred and she died in the 51st week after the first injection.

The effect on leucocytes was of particular interest. The number of leucocytes at the start of treatment was 3000, dropped to 1700 in the 9th week after the first injection of Thio-TEPA and quickly rose to 7100 when clinical improvement commenced.

Time of observation was 51 weeks.

CASE 2 (FIG. 2)

Age 56. Histology : well differentiated, probably granulosa cell carcinoma. First symptom : April 1956, swelling of lower abdomen.

FIG. 2

Degree of clinical improvement – – – – – – – – – – –

Grade 1. Improvement of symptoms.
2. Disappearance of symptoms.
3. Decrease in size of tumour or decrease of ascites.
4. Clinical disappearance of ascites.
5. Disappearance of tumour, of ascites and of symptoms.

Normal activity 

Restricted activity 

13.8.56 subtotal hysterectomy and bilateral oophorectomy for bilateral ovarian carcinoma. Secondary nodules on pelvic peritoneum and peritoneum overlying the bladder. 13.8.56 2 pints of blood transfused.

September/October 1956 post-operative X-ray treatment and one radium application to uterus.

Prior to Thio-TEPA : pelvic pain, abdominal distension and bleeding from the umbilicus on slight trauma. Examination after removal of 3000 ml. of ascites by paracentesis on 12.11.57 : nodular mass in pouch of Douglas extending into both sides of pelvis. Secondary tumour, 2 cm. diameter, in umbilicus.

Commencing on 13th November 1957, she was given the first course of Thio-TEPA, 3 intra-peritoneal injections of 25 mg. each, in 3 weeks. She vomited occasionally during the first 7 days ; from the second week onwards she gained strength, her appetite improved and she felt well. From the 8th week onwards there was no evidence of disease and she was free from symptoms. Definite decrease in size of the tumour was observed in the 7th week after the first injection and in the 11th week all growth and ascites had disappeared. She led a normal life doing all her housework for approximately 8 months, then pain, ascites, tumour of pelvis and umbilicus reappeared rapidly.

She was given further injections of Thio-TEPA commencing with a course of 4 injections of 25 mg. each in 4 weeks, and later further injections as shown in Fig. 2.

Six weeks after the start of the second course she again gained in strength, attained a sense of well-being and her appetite improved ; her symptoms disappeared completely and she returned to her normal activities for $3\frac{1}{2}$ months. During this period ascites disappeared, the growth had decreased in size but did not disappear ; then pain and ascites recurred rapidly and frequent paracentesis became necessary. Further injections were given as shown in the graph.

The patient died 19.8.59, 92 weeks after the first injection of Thio-TEPA.

### CASE 3 (FIG. 3)

Age 44. Histology : granulosa cell carcinoma producing pseudoadenomatous appearances.

First symptoms : March 1958, pain in left abdomen.

Operation : 24.9.58 : gross ascites, abdomen filled with bilateral multilocular ovarian cystic tumours with a large amount of solid and friable tissue. Metastases on pelvic peritoneum and uterus. Both ovarian new growths removed.

Prior to Thio-TEPA : 27.10.58 ascites ; firm mass of growth in upper third of rectovaginal septum and pouch of Douglas ; large mass in left pelvis and a large mass in left lumbar region extending into left hypochondrium.

Treatment with Thio-TEPA commenced on 10.11.58 : a course of 84 mg. was given in 5 injections in 10 days, and a second course of 116 mg. commenced 8 weeks after the first injection.

From the 10th day onwards she gained strength, her appetite improved and later on she felt well and was doing light housework except when ascites had accumulated. From the 14th week onwards tumour decreased in size but did not disappear. In the 19th week a paracentesis of abdomen was necessary, subsequently she was well and doing light housework until the 25th week when ascites and symptoms recurred. Further injections were given as shown in Fig. 3.

The patient died 8.10.59, 47 weeks after the first injection of Thio-TEPA.

### CASE 4 (FIG. 4)

Age 52. Histology : serous papillary adenocarcinoma of ovary.

First symptom : May 1958, swelling of abdomen.

Operation 13.8.58 : 4500 ml. of ascitic fluid removed. Right ovarian cystic tumour of 12 cm. diameter removed. Peritoneal metastases present.

13.8.58 : 4 pints of blood transfused.

Fig. 3

Degree of clinical improvement - - - - - - - - - - -

Grade 1. Improvement of symptoms.
2. Disappearance of symptoms.
3. Decrease in size of tumour or decrease of ascites.
4. Clinical disappearance of ascites.
5. Disappearance of tumour, of ascites and of symptoms.

Normal activity

Restricted activity

Post-operative X-ray treatment September/October 1958, could not be completed because of poor general condition.

Prior to Thio-TEPA : November 1958, examination under anaesthesia after removal of 5700 ml. of ascites : a mass consisting of matted spherical tumours filling pelvis and lower abdomen extending into upper abdomen on the right side.

Main symptoms : lassitude and gnawing abdominal pain.

On 22.11.58 the first course of Thio-TEPA commenced ; 100 mg. were given in 4 intra-peritoneal injections of 25 mg. each at weekly intervals, and she was given a further 25 mg. in the 16th and 17th week after the first injection.

In the 6th week decrease in size of tumour mass was observed, and in the 10th week the tumours had disappeared except for one 4 cm. diameter lump at the

Fig. 4

Degree of clinical improvement — — — — — — — — — — —

Grade 1. Improvement of symptoms.
2. Disappearance of symptoms.
3. Decrease in size of tumour or decrease of ascites.
4. Clinical disappearance of ascites.
5. Disappearance of tumour, of ascites and of symptoms.

Normal activity 

Restricted activity 

level of umbilicus which was still palpable. At that time ascites had disappeared and did not re-accumulate during the time of observation. The lump at the level of umbilicus continued to decrease in size but did not completely disappear. From the second week onwards her appetite improved, she gained strength and attained a sense of well-being, and from the 18th week onwards she could do all her housework, and was enjoying a normal life.

She remained well and vigorous and the tumours decreased further in size until August 1959. In August the tumours, ascites and symptoms reappeared.

A further 3 intra-peritoneal injections of 30 mg. each were given followed by improvement of symptoms and return to her activity for 8 weeks. Since then the growth increased in size, she lost strength and flesh rapidly.

CASE 5 (FIG. 5)

Age 55. Histology : granulosa cell carcinoma.
First symptom : December 1955, malaise.
13.1.56 : removal of a large left multilocular cystic ovarian growth.

FIG. 5

Degree of clinical improvement - - - - - - - - - - - -
Grade 1. Improvement of symptoms.
2. Disappearance of symptoms.
3. Decrease in size of tumour or decrease of ascites.
4. Clinical disappearance of ascites.
5. Disappearance of tumour, of ascites and of symptoms.

Normal activity
Restricted activity

May 1957 : large fixed hard nodular mass of growth in pouch of Douglas and left pelvis. X-ray treatment and one radium application to uterus was followed by only slight regression of growth and temporary alleviation of pain lasting approximately 6 months.

Prior to treatment with Thio-TEPA : January 1959, extensive growth involving rectum and rectovaginal wall causing stenosis of rectum, oedema of legs, vulva and buttocks and gross distension of veins of abdomen. She had nausea, occasional vomiting, anorexia, frequent watery stools and sometimes loss of control, frequency of micturition, abdominal pain and tiredness even at rest : she was still doing a little light housework.

Treatment with Thio-TEPA started on 27.1.59. She was given a course of 6 injections to a total of 100 mg. in 3 weeks and then one 27 mg. intra-muscularly 9 weeks after the first injection.

From the second week onwards pain and nausea decreased, appetite improved and she gained a little in strength.

The tumour decreased in size slightly and the oedema of legs, vulva, buttocks and the distension of abdominal veins disappeared. A rectovaginal fistual developed in the 12th week. The distension of abdominal veins and oedema of thighs and pain reappeared in the 14th week.

A colostomy was done and one pint of blood was transfused on 4.6.59.

The patient died 9.6.59 ; 19 weeks after the first injection of Thio-TEPA.

CASE 6 (FIG. 6)

Age 54. Histology : papilliferous cystadenocarcinoma.

First symptom : May 1956, pain in right lower abdomen.

15.9.58 : complete removal of left ovarian growth, incomplete removal of large friable growth of right ovary which was adherent to uterus and pelvic wall.

17.9.58 : 2 pints of blood transfused.

October/December 1958 : one radium application to uterus and X-ray treatment to pelvis.

Prior to treatment with Thio-TEPA : a large mass in the pelvis involving uterus pressing on rectum. Constant pain in abdomen and in right shoulder for which she required alternatively papaveretum gr. $\frac{1}{3}$ or pethidine 100 mg. 4 hourly day and night. She was very ill and confined to bed.

5.12.58 ; 2 pints of blood transfused.

Treatment with Thio-TEPA commenced on 10.2.59.

She was given at first 62·5 mg. in 7 intra-venous injections in 17 days.

From the 6th week onwards her appetite improved, she gained strength and pain decreased. Decrease in size of the tumour commenced in the 8th week ; in the 9th week abdominal pain had almost completely disappeared. She was discharged home and in the 12th week she could walk around and help a little in her household.

A further 2 intra-muscular injections of 30 mg. of Thio-TEPA were given in December. Objective and subjective improvement was maintained.

CASE 7 (FIG. 7)

Age 54. Histology : papillary carcinoma of ovary.

First symptom : October 1958, lassitude.

12.11.58 operations : gross ascites. Omentum and peritoneum studded with nodules, pelvis filled with growth. During the 2 months preceeding the first injection of Thio-TEPA she needed paracentesis on 6 occasions at intervals of 5 to 21 days.

Prior to Thio-TEPA: gross distension of abdomen due to large masses of growth and ascites; loss of flesh; lassitude, vomiting, swelling of legs when abdomen distended by ascites.

On 12.2.59 treatment with Thio-TEPA commenced. She was given as the first course 150 mg. divided into 5 intra-peritoneal injections at weekly intervals. Further injections as shown in Fig. 7.

FIG. 6

Degree of clinical improvement – – – – – – – – – – – –

Grade 1. Improvement of symptoms.
2. Disappearance of symptoms.
3. Decrease in size of tumour or decrease of ascites.
4. Clinical disappearance of ascites.
5. Disappearance of tumour, of ascites and of symptoms.

Normal activity

Restricted activity

During the first 2 weeks after the first injection a paracentesis was done on two occasions. From the 3rd week onwards ascites decreased slightly. She gained strength, her appetite improved and she felt well.

She is doing her shopping and working part time as a typist.

Change in the size of the tumour is difficult to assess because of ascites.

The time of observation since the first injection of Thio-TEPA was 15 weeks on June 1st 1959.

From June 1st till October 9th 1959 she was given a further 140 mg. in 6 intra-peritoneal injections. Paracentesis abdominis was necessary once in October 1959.

Fig. 7

Degree of clinical improvement – – – – – – – – – – – –

Grade 1. Improvement of symptoms.
2. Disappearance of symptoms.
3. Decrease in size of tumour or decrease of ascites.
4. Clinical disappearance of ascites.
5. Disappearance of tumour, of ascites and of symptoms.

Normal activity 

Restricted activity 

At the end of December ascites and abdominal tumour were present; she felt quite well except at the time when a paracentesis had become necessary; she continues doing part time work as a typist and also doing light housework.

CASE 8

Age 55. No histology.
First symptom: December 1958, swelling of abdomen.
Prior to treatment with Thio-TEPA: distension of abdomen, shortness of breath, loss of weight and anorexia. A paracentesis of chest and abdomen was done 5 times during the preceding 2 months.

Examination after removal of 7 litre of fluid by paracentesis of abdomen : large nodular growth in pelvis filling pouch of Douglas extending upwards to the level of umbilicus. Multiple large lobulated tumour masses in abdomen. Liver enlarged.

Treatment with Thio-TEPA commenced on 18.3.59. She was given 4 intra-peritoneal injections at weekly intervals to a total of 120 mg. Ascites was removed by paracentesis on 4 occasions during the 6 weeks following the first injection. From the 5th week onwards she gained strength and her appetite improved.

In the 9th week ascites and pleural effusion increased causing dyspnoea and therefore fluid was removed by paracentesis of chest.

The patient died on 20.7.59, 18 weeks after the first injection of Thio-TEPA.

### CASE 9

Age 45. Histology : granulosa cell carcinoma.

First symptom : November 1956, pain in left pelvis.

March 1957 : hysterectomy and bilateral salpingo-oophorectomy for carcinoma of ovary.

April/May 1957 : post-operative X-ray treatment to pelvis.

July 1958, secondary node in operation scar treated with X-ray.

Mass of growth in pelvis treated with X-ray November/December 1958.

Prior to treatment with Thio-TEPA : a fixed hard mass in left pelvis extending almost to the level of umbilicus. 4 cm. diameter subcutaneous metastatic tumour in abdominal wall in upper end of operation scar. Liver enlarged. Slight pain in right iliac fossa, slight backache and nausea.

Treatment with Thio-TEPA commenced on 18.3.59. She was given at first 3 injections intra-muscularly of 25 mg. each at weekly intervals.

The leucocytes dropped from 6400 to 2300 in the 3rd week ; a further 25 mg. was given after the leucocytes had risen to 4200 at the end of the 6th week.

From the second week onwards she gained strength and attained a sense of well-being. No definite change in the size of the tumour has been observed.

The patient died 4.11.59, 30 weeks after the first injection of Thio-TEPA.

### METHOD

The crystalline Thio-TEPA was dissolved in normal saline 10 mg. per ml. and stored no longer than 14 days in refrigeration. The route and dose in this series varied. If ascites was present the injections were given into the peritoneal cavity. A small quantity of 2 per cent procaine was injected locally before giving Thio-TEPA intra-muscularly or into the tumour. The dose varied from case to case depending on the patients strength, the state of nutrition, haematological status and on the route. From our experience we came to the conclusion that 65 to 120 mg. given in fractionated doses over three to four weeks is an effective therapeutic dose. Intravenously Thio-TEPA was given at the rate of 7 to 10 mg. per day to a weekly maximum dose of 30 mg. Injections of Thio-TEPA are discontinued when the graph of total leucocytes showed a downward trend following previous injections.

Principles of maintenance treatment are yet to be developed. We intend to give a second course in all cases which show a good response, at the latest 3 to 5 months after the first course.

It is of great value that most cases can be treated as out patients.

### EFFECT AND REACTIONS

In 4 of our cases of advanced carcinoma of the ovary, treatment with Thio-TEPA was followed by striking temporary regression or clinical disappearance of the growth, reduction or disappearance of ascites and marked improvement of symptoms with return to activity and enjoyment of life. Three patients had definite marked temporary regression of growth and improvement of symptoms only. The maximum duration of improvement after one course was 8 months. Second and subsequent courses were less effective than the first course.

Improvement in strength, appetite and sense of well-being, soon after the start of treatment, was noted in all cases in whom Thio-TEPA had any clinical effect.

Though symptoms may improve during or shortly after the second week, decrease in size of tumour and of ascites occurs mostly after 6 to 8 weeks.

All our cases who showed a marked improvement had temporary leukopenia following treatment.

Decrease in the number of granulocytes and platelets commenced in the second or third week, and recovered quickly to normal. Petechiae and cutaneous haemorrhages were observed in two cases at the time of maximum depression of haemopoiesis and subsided spontaneously.

The effect of Thio-TEPA on leucocytes was remarkable in case 1 in whom the number of leucocytes at the start of treatment was 3000, dropped to 1700 8 weeks after treatment and rose to 7100 one week later. The fall and later the rise of the number of leucocytes coincided with retrogression of growth and improvement of symptoms.

Only one case had side reactions such as nausea and vomiting. There were no unpleasant local or general reactions in any of the other cases.

### DISCUSSION

Thio-TEPA has a marked growth inhibiting effect in some cases of carcinoma of the ovary. The effect is only temporary. Thio-TEPA does not supersede surgery or radiotherapy in the treatment of ovarian cancer. It is, however, of definite value in some cases in whom all other treatments have failed. In such cases Thio-TEPA may still achieve temporary control of disease to such an extent that the patient may enjoy a normal life and may return to normal activity for many months. The duration of temporary control will, one can reasonably expect, increase with greater experience and by the various means of protecting the haemopoietic tissue.

Small repeated transfusions of blood given daily for a few days before, and during treatment with Thio-TEPA, may reduce the proliferative and mitotic activity of haemopoietic tissue to a resting phase. In the resting phase the bone marrow may be less sensitive to a radio-mimetic and antimitotic agent.

If this concept is correct, 50 ml. of blood given daily should reduce the bone marrow activity. A plan for investigation in this direction is in preparation.

From what has already been done in this field, and from our own experience, we conclude that ovarian carcinoma occupies the foremost position in the spectrum of activity of Thio-TEPA over the whole range of malignant disease.

In many cases of advanced cancer classification by histology is often difficult and sometimes debatable. It is likely that some cases which appear to be primary malignant tumours may, in fact, be secondaries from a growth elsewhere. If this is true the selective effect of Thio-TEPA on primary ovarian carcinoma is probably higher than it appears from our series. It is of immediate interest to correlate sensitivity of the various groups of ovarian cancer to chemotherapeutic agents with their natural history and histology, and to study each group according to their sensitivity.

A plan of study of the effectiveness of chemotherapy as an adjuvant to surgical treatment of cancer has been described by Shimkin and Moore (1958).

Two investigations are being carried out using Thio-TEPA in resectable carcinoma of stomach and nitrogen mustard in resectable cancer of lung. One of the aims of the plan is to ascertain whether chemotherapeutic agents are more effective when the tumours are small and clinically not obviously established.

The discovery of the effect of Thio-TEPA on carcinoma of ovary has opened new and promising paths in the field of experimental and clinical research on ovarian carcinoma.

### SUMMARY

The effect of Thio-TEPA in 17 cases of advanced carcinoma has been described.

Objective improvement has been achieved in 8 cases, some of whom have returned to their normal activity for several months. The clinical effect is temporary. In all cases the effect was associated with transient leukopenia.

We are most grateful to the Lederle Laboratories, Division of Cyanamid of Great Britain Limited, for their generous supply of Thio-TEPA and for literature and references.

We thank Mrs. Sheila Cocks, M.S.R., Superintendent Radiographer, and the Photographer, Mr. Brooks, for the reproduction of the graphs.

We also thank Miss Pam Martin for her secretarial help.

### REFERENCES

ALPERT, L. K.—(1958) *Ann. N.Y. Acad. Sci.*, **68**, 1072.

BATEMAN, J. C.—(1958) *Ibid.*, **68**, 1057.—(1955) *New Engl. J. Med.*, **252**, 879.

LEONARD, B. J., ISRAELS, M. C. G. AND WILKINSON, J.—(1956) *Lancet*, ii, 1017.

LEONE, L. A.—(1958) *Ann. N.Y. Acad. Sci.*, **68**, 1081.

MOORE, G. E.—(1958) *Ibid.*, **68**, 1074.

OLSON, K. B.—(1958) *Ibid.*, **68**, 1018.

SHAY, H., LARAFONETIS, C., SMITH, N., WELDOW, I. and SUN, C. H.—(1953) *Arch. intern. Med.*, **92**, 628.

*Idem* AND SUN, C. H.—(1958) *Ann. N.Y. Acad. Sci.*, **68**, 1046.

SHIMKIN, M. B. AND MOORE, G. E.—(1958) *J. Amer. med. Ass.*, **167**, 1710.

ULTMAN, J. E., HAYMAN, G. A. AND GELLHORN, A.—(1958) *Ann. N.Y. Acad. Sci.*, **68**, 1007.

WATSON, G. W. AND TURNER, R. L.—(1959) *Brit. med. J.*, i, 1315.

WRIGHT, J. C., GOLOMB, F. M. AND GUMPORT, S. L.—(1958) *Ann. N.Y. Acad. Sci.*, **68**, 937.